# HIDEA syndrome: A new case report highlighting similarities with ROHHAD syndrome

**DOI:** 10.3389/fgene.2023.1137767

**Published:** 2023-03-22

**Authors:** J. Harvengt, A. Lumaka, C. Fasquelle, J. H. Caberg, M. Mastouri, A. Janssen, L. Palmeira, V. Bours

**Affiliations:** ^1^ Human Genetics Department, CHU of Liège, Liège, Belgium; ^2^ GIGA Research, University of Liège, Liège, Belgium; ^3^ Pediatric Department, Hospital Center of Luxembourg, Luxembourg City, Luxembourg; ^4^ Pediatric Department, CHU of Liège, Liège, Belgium

**Keywords:** ROHHAD, rapid-onset obesity with hypoventilation, hypothalamic dysfunction and autonomic dysregulation, central hypoventilation, HIDEA syndrome, *P4HTM*, childhood obesity

## Abstract

**Context:** ROHHAD syndrome presents a significant resemblance to HIDEA syndrome. The latter is caused by biallelic loss-of-function variants in the *P4HTM* gene and encompasses hypotonia, intellectual disabilities, eye abnormalities, hypoventilation, and dysautonomia. We report the first patient identified with HIDEA syndrome from our ROHHAD cohort.

**Clinical case:** Our patient was a 21-month-old girl who had a history of severe respiratory infections requiring intensive care, hypotonia, abnormal eye movements, and rapid weight gain. Polysomnography identified severe central hypoventilation. During her follow-up, a significant psychomotor delay and the absence of language were gradually observed. The prolactin levels were initially increased. Hypothermia was reported at 4 years. Exome sequencing identified a new homozygous truncating *P4HTM* variant.

**Discussion:** Our patient met the diagnosis criteria for ROHHAD, which included rapid weight gain, central hypoventilation appearing after 1.5 years of age, hyperprolactinemia suggesting hypothalamic dysfunction, and autonomic dysfunction manifesting as strabismus and hypothermia. However, she also presented with severe neurodevelopmental delay, which is not a classic feature of ROHHAD syndrome. HIDEA syndrome presents similarities with ROHHAD, including hypoventilation, obesity, and dysautonomia. To date, only 14% of endocrinological disturbances have been reported in HIDEA patients. Better delineation of both syndromes is required to investigate the eventual involvement of *P4HTM*, a regulator of calcium dynamics and gliotransmission, in ROHHAD patients.

**Conclusion:** In the case of clinical evidence of ROHHAD in a child with abnormal neurological development or eye abnormalities, we suggest that the *P4HTM* gene be systematically interrogated in addition to the analysis of the *PHOX2B* gene. A better delineation of the natural history of HIDEA is required to allow further comparisons between features of HIDEA and ROHHAD. The clinical similarities could potentially orient some molecular hypotheses in the field of ROHHAD research.

## Introduction

Rapid-onset obesity with hypothalamic dysfunction, hypoventilation, autonomic dysregulation, and neural crest tumor [ROHHAD (NET)] is a rare and potentially fatal disease for which a specific diagnostic biomarker is currently unavailable. The definition of ROHHAD syndrome is currently based on clinical criteria, defined first by Ize-Ludlow et al. (2007). The major criterion is dramatic weight gain associated with central hypoventilation appearing between 1.5 and 7 years of age. This rapid-onset obesity is considered to be the first sign of hypothalamic dysfunction. At least one other sign of hypothalamic dysfunction is necessary for its diagnosis, such as hyperprolactinemia, central hypothyroidism, disordered water balance, abnormal growth hormone (GH) response, adrenocortical insufficiency, or puberty disorders. Central hypoventilation can occur rapidly after the initial weight gain or can appear during the following years (83% were reported to have been diagnosed with hypoventilation after a maximum of 5 years of follow-up). This autonomic dysfunction may also manifest later with other dysregulations such as thermal dysregulation, excessive sweating, cardiovascular manifestations (arrhythmias or blood pressure dysregulation), strabismus, abnormal pupillary reaction to light, or gastrointestinal or sensory disturbances.

In cases of patients presenting with rapid weight gain, hypoventilation, and hypothalamic dysfunction, an early diagnosis of ROHHAD has to be considered to improve management and prognosis. Nevertheless, at the same time, etiological investigations need to be extended to explore rare differential diagnosis (Harvengt et al., 2020). The current recommendations include the systematic screening of ROHHAD patients for *PHOX2B* gene disorder to rule out congenital central hypoventilation syndrome. However, the wider availability of whole exome sequencing (WES) analyses tends to show new results in patients who have been initially investigated for clinical signs that may partly overlap with those of ROHHAD syndrome.

We report here, for the first time, the case of a patient encompassing some ROHHAD criteria in whom HIDEA syndrome has been identified. HIDEA (OMIM #618493), first described in 2019, is an acronym for hypotonia, hypoventilation, impaired intellectual development, dysautonomia, epilepsy, and eye abnormalities.

## Clinical case

**FIGURE 1:**
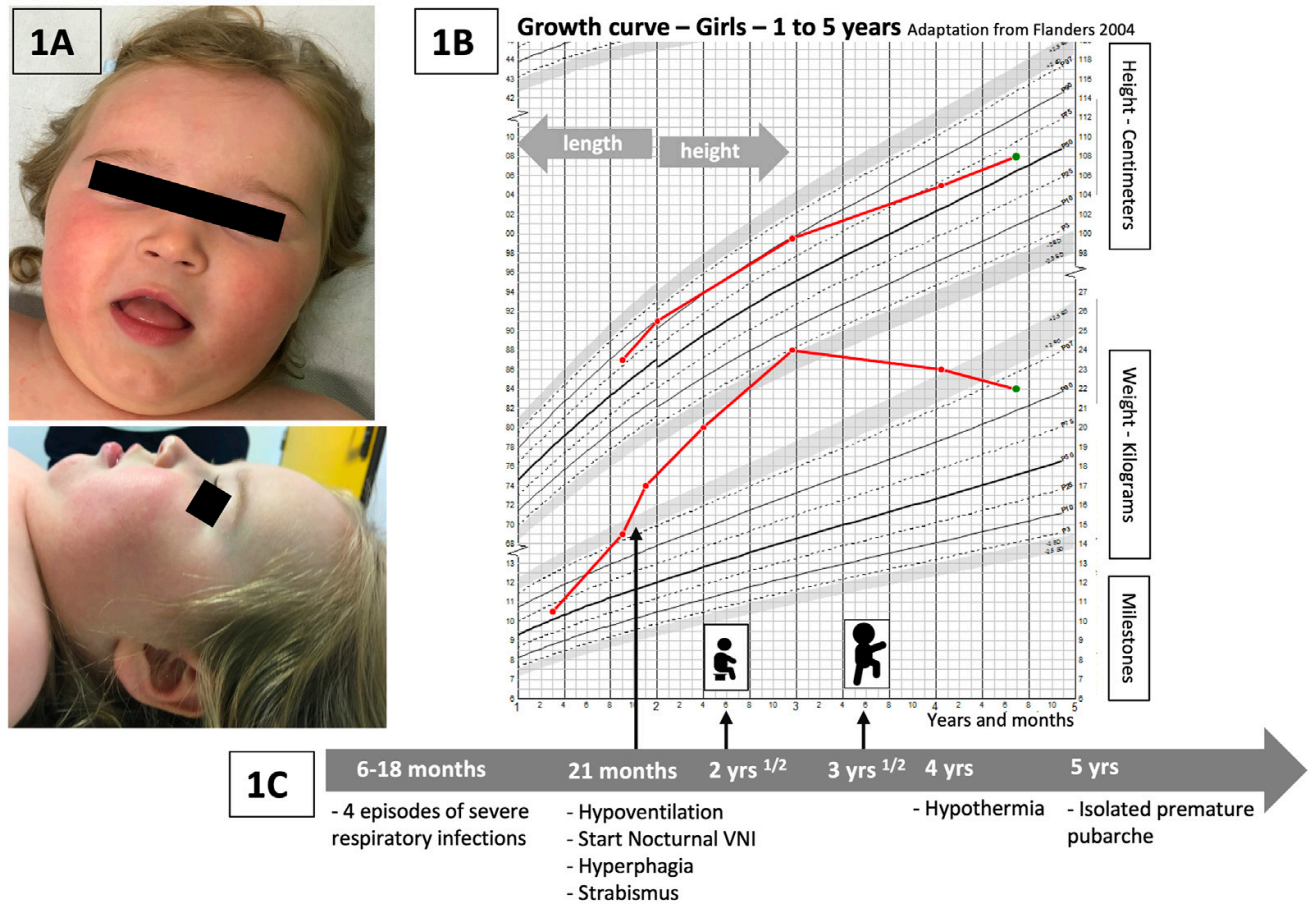
**(A)** Pictures of the patient: face and profile. No specific dysmorphism is discriminated, but similarities with previous publications are encountered: global facial hypotonia, tented upper lip vermilion, and a low nasal bridge (Kraatari-Tiri et al., 2022). **(B)** Weight and height curves of our patient (growth chart—Flanders, 2004, for girls, 1–5 years). Rapid weight gain starting just before 2 years, significant increase between 2 and 3 years of age, and progressive improvement and stabilization of weight related to height at around 4 years of age. **(C)** Timeline with the main relevant clinical data of the follow-up of our patient.

Our patient (Figure 1A) is a girl who was initially hospitalized in the context of persistent hypercapnia at 21 months of age. Her neonatal history revealed a prematurity of 35 weeks of gestation, BW 2.8 kg (0.13 SD) and BL 48 cm (2.2 SD) but otherwise the pregnancy, delivery and neonatal period had been uneventful. Her parents were healthy and unrelated with no particular family history. Between the ages of 6 and 18 months, she presented with four severe viral respiratory infections [bronchiolitis, one episode due to an RSV (respiratory syncytial virus)] requiring intensive care and invasive ventilation for three of these episodes. The fifth infectious episode occurred at 21 months of age, and persistent hypercapnia was observed with confirmation of central hypoventilation on the polysomnography record. Nocturnal non-invasive ventilation was started and well tolerated. At the same time, she presented with rapid weight gain (Figure 1B). Her parents reported a total lack of satiety. Hormonal workup showed initially increased prolactin levels and no hypothyroidism. IGF1 dosages were slightly increased (340 ng/mL). The initial evaluation was complemented by an ophthalmologic assessment because of abnormal eye movements. Severe strabismus was confirmed but with no other retinal abnormalities. Cardiac ultrasound and electrocardiogram were normal. Global hypotonia was observed during hospitalizations, more severely at 21 months of age, with poor postural control. Cerebral MRI was normal.

During her follow-up, severe psychomotor delay was progressively noted. She sat at 2.5 years and walked at 3.5 years (Figure 1C). She developed no language before the age of 4 years and only a few short words at 6 years. The swallowing function was also delayed; however, she could eat solid food and drink liquids with assistance at the age of 6 years. At 4 years of age, hypothermia was observed with a temperature measurement of 34°C without particular symptoms (the temperature measurement was taken daily during the COVID-19 pandemic in her school institution, which led to the incidental detection of the hypothermia episodes).

Currently, she has no signs of epilepsy. The weight gain is being progressively controlled with a healthy diet and the stimulation of limited physical activities (she walks with assistance). The respiratory function is relatively well controlled with nocturnal non-invasive ventilation, with only one recent acute respiratory decompensation at 6 years. Notably, she presented with an isolated premature pubarche starting at the age of 5.5 years.

**FIGURE 2:**
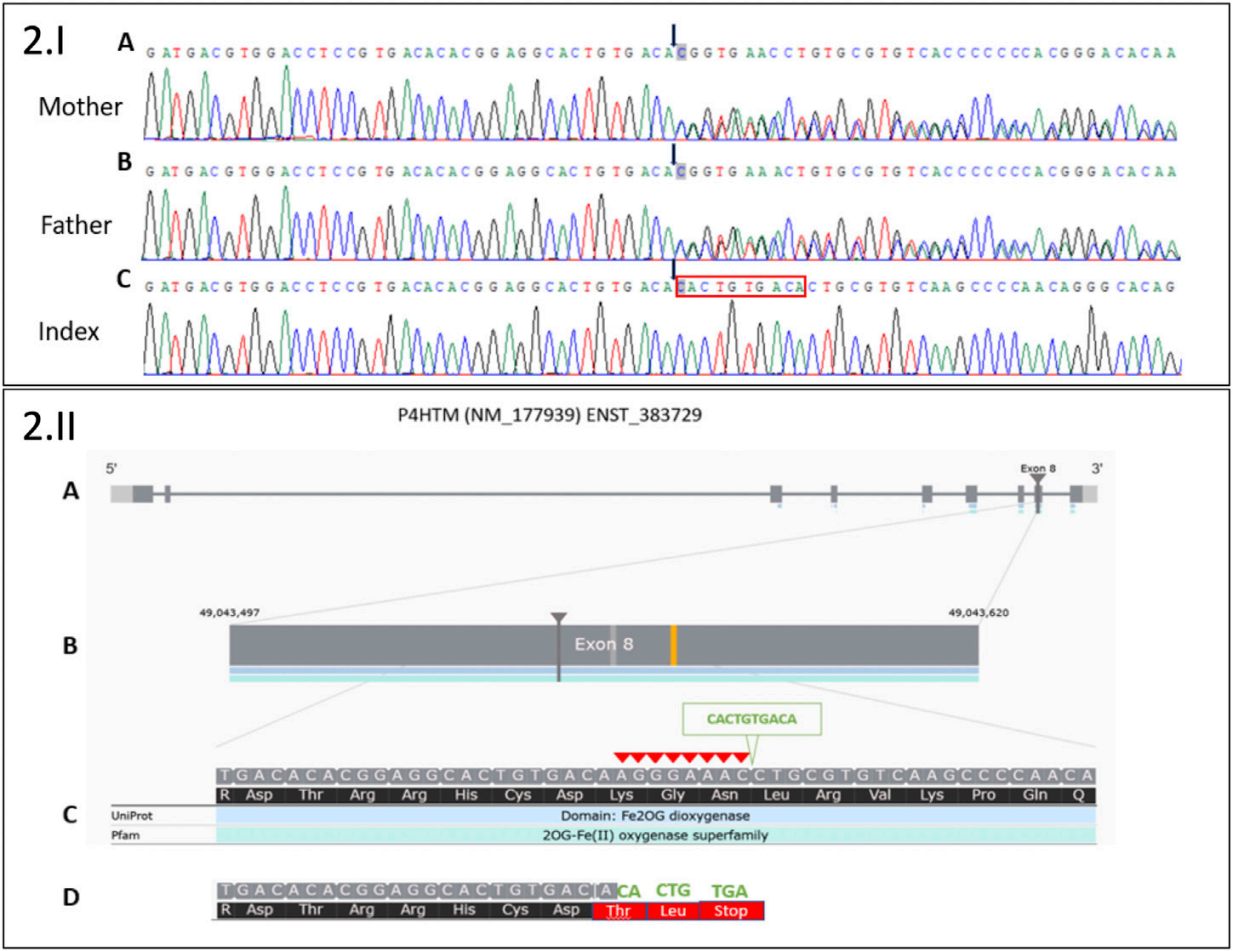
2.I Sanger sequencing electropherograms of a novel deletion 8 bp/insertion 10 bp in exon 8 of the *P4HTM* gene: **(A)** in the heterozygous state in the healthy mother, **(B)** in the heterozygous state in the healthy father, and **(C)** in the homozygous state in the index. Black arrows indicate the start of 8 bp deletion (AGGGAAAC)/10 bp insertion (CACTGTGACA). The red box indicates the 10 bp insertion in Index. 2.II. Moon visualization of the *P4HTM* gene. **(A)** Transcript version NM_177939 of *P4HTM* gene with nine coding exons. **(B)** The deletion (8 bp)/insertion (10 bp) mutation is located in exon 8 of the transcript. **(C)** Red arrowheads indicate 8 bp deletion (AGGGAAAC). The green arrowhead indicates 10 bp insertion (CACTGTGACA). **(D)** The substitution creates a premature stop codon (three codons downstream): NM_177939.3: c.1217_1224delinsCACTGTGACA; NP_808808.1:p.(Lys406Thrfs*3).

Molecular investigations first encompassed *PHOX2B* gene analysis, molecular karyotyping, and Prader–Willi syndrome analysis. All of these were normal. A WES-filtered panel dedicated to intellectual disabilities initially identified no pathogenic variant. Because of the patient's clinical evolution, at 5 years of age, the whole exome analysis was requested, and a new homozygous pathogenic variant in the *P4HTM* gene was identified, providing the diagnosis of HIDEA syndrome (Figure 2).

## Methods

### Molecular investigations

Trio-based exome (in the proband and her unaffected parents) was performed at the Human Genetics Laboratory, CHU of Liège. Genomic DNA was extracted from whole blood with the NucleoMag^®^ Blood 200 μL kit (Macherey-Nagel) automated on a STARlet platform (Hamilton) following the manufacturer’s instructions. DNA concentrations and quality were measured using the Qubit^®^ DNA Assay Kit in a Qubit^®^ 2.0 Fluorometer (Invitrogen) and agarose gel electrophoresis. A total of 1.0 μg genomic DNA per sample was used as the input material for the exome library preparation. The exome was captured using the Agilent SureSelect Human All Exon V6 following the manufacturer’s recommendations. Sequencing was performed using the Illumina NovaSeq 6000 system in paired-end (2 × 150 cycles). The average depths of coverage obtained were 24× for the proband, 21× for the healthy father, and 35× for the healthy mother.

The raw results were aligned to the reference genome (GRCh37/hg19) using a homemade pipeline (Humanomics v2.0) that utilizes published algorithms in a sequential manner according to the GATK v3.8 toolkit: BWA-MEM v0.7.17 for mapping the reads, Picard v2.20 for marking the end and removing optical and PCR duplicates, HaplotypeCaller for detecting variants, and GenotypeGVCFs for producing VCF files. VCF files are used for the interpretation of variants using the Moon software from Invitae. Genome Reference Consortium Human Build 38 (GRCh38/hg38) was used as the genome assembly reference.

Several variants were detected in exon 8 of the *P4HTM* gene, suggesting an indel in this region. PCR was performed on genomic DNA to amplify exon 8 of the *P4HTM* gene with the following primers: 5′CTC​ACC​TCT​CCC​CAC​AAG​TT3′ and 5′GGG​CAG​ATG​GAG​TCA​GTA​CA3′ (PCR product size, 1,906 bp). Sanger sequencing was performed on the PCR product with the same primers. The electropherogram analysis revealed deletion (8 bp)/insertion (10 bp) in the heterozygous state for the healthy parents and in the homozygous state for the patient. The deletion (in-frame deletion) of 8 bp (AGGGAAAC) was compensated by the substitution of 10 bp (CACTGTGACA). The substitution created three stop codons downstream: NM_177939.3: c.1217_1224delinsCACTGTGACA; NP_808808.1:p.(Lys406Thrfs*3). According to the current ACMG classification guidelines, this result is considered a class 4 variant (PVS1 and PM2 positive criteria).

## Discussion

**FIGURE 3:**
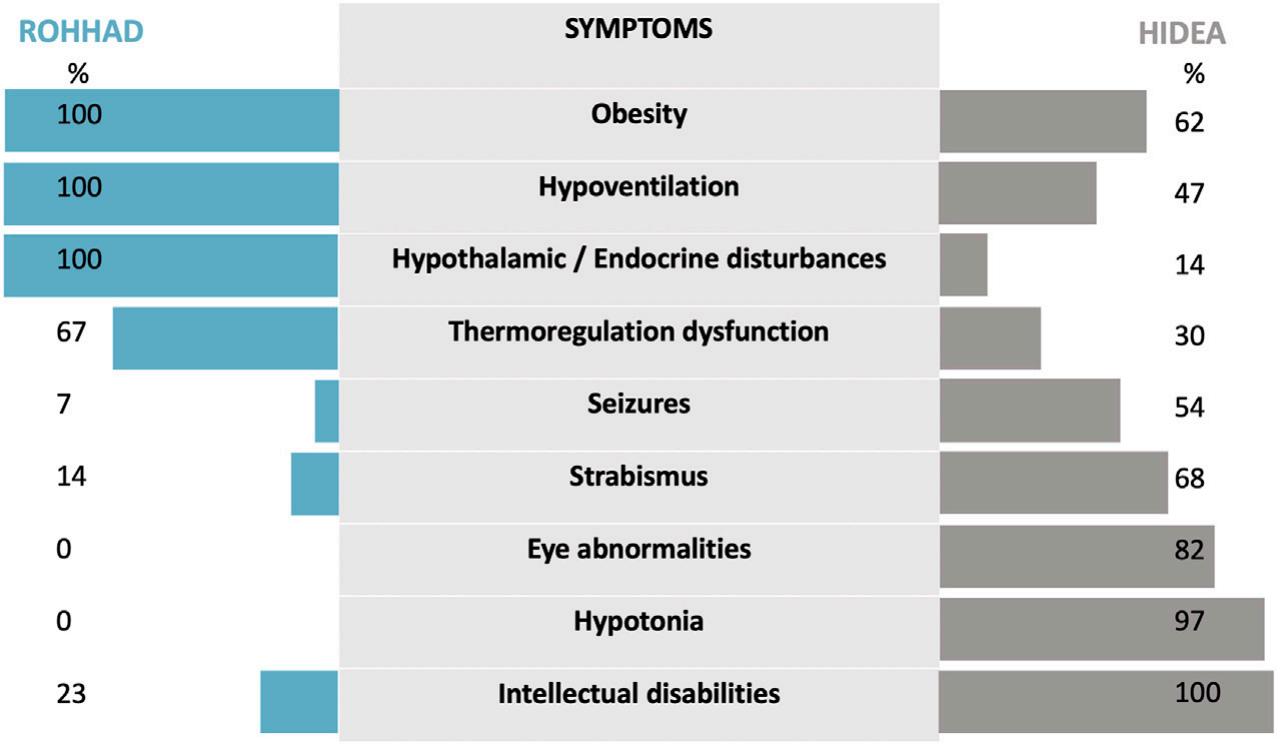
The phenotypic features of HIDEA patients demonstrate an overlap with ROHHAD characteristics. For HIDEA: percentages based on the data of 31 patients—our patient and 30 patients—described in the review of Kraatari-Tiri et al. (2022). [which included 24 previously reported patients (Kaasinen et al., 2014; Rahikkala et al., 2019; Maddirevula et al., 2020; Hay et al., 2021; Järvelä et al., 2021; Lim et al., 2022) and six new patients]. For ROHHAD: percentages are based on the data from Harvengt et al. (2020).

This report highlights for the first time the clinical similarities between HIDEA and ROHHAD syndromes. Few data regarding HIDEA are currently available in the literature. A recent cohort description has been published describing 24 previously reported patients and 6 new patients (Rahikkala et al., 2019; Maddirevula et al., 2020; Hay et al., 2021; Kraatari-Tiri et al., 2022). A comparison with a ROHHAD descriptive cohort of 44 patients (Harvengt et al., 2020) was made (Figure 3). The phenotypic features of the HIDEA patients demonstrated an overlap with the ROHHAD characteristics.

First, obesity and central hypoventilation are the initial symptoms of both syndromes. A ROHHAD diagnosis is based on clinical criteria, and the lack of a biological marker makes it difficult for the clinician to rule out the diagnosis in a young patient with central hypoventilation and rapid weight gain. The symptoms in ROHHAD typically (but not categorically) begin in a child with no previous specific medical history (Harvengt et al., 2020). One major factor in discriminating the diagnoses of both ROHHAD and HIDEA is carefully following the clinical evolution of the patient during the few months following the initial symptoms. This is especially illustrated by patients such as ours for whom during the initial diagnosis investigation phase, no evident argument could distinguish between the two hypotheses of ROHHAD and HIDEA. Regarding hypoventilation, successive hospitalizations for severe bronchiolitis were probably due to the decompensation of untreated chronic hypoventilation. The other clinical signs presented by our patient could also be clear arguments for the HIDEA diagnosis but only on retrospective analysis. Our patient presented progressive hypotonia and motor delay. It was not clear at the time whether the hypotonia was related to a syndromic disorder or to poor socio-educational conditions. Molecular analysis was, therefore, considered to be the most appropriate way in this case to investigate the differential diagnoses of ROHHAD. A panel of genes dedicated to intellectual disabilities was initially negative. The analysis was extended to the WES to finally establish the diagnosis of HIDEA syndrome.

Dysautonomia is a second similar feature. It is an essential clinical sign encountered in ROHHAD patients that has been reported in 30% of HIDEA patients through thermal dysregulation. Consequently, it is not clear whether cardiac rhythm alterations, sweating alterations, or digestive dysmotility could be part of this syndrome. Strabismus has been reported in 68% of HIDEA patients, and we do not know whether strabismus is an independent symptom that is related to neurological dysfunction or whether it could be a part of the dysautonomia process. In ROHHAD patients, strabismus is only described as a part of the dysautonomia symptoms in a total of 6/44 patients but has not been excluded as a symptom that could be more frequent in ROHHAD, considering frequent incomplete case reports (Harvengt et al., 2020).

As the last point of comparison, hypothalamic or endocrine disturbances have been reported for only 14% of HIDEA patients. The 14% includes a total of three patients: one with precocious adrenarche (Rahikkala et al., 2019), one with hypothyroidism (Maddirevula et al., 2020), and one (the subject of our case report) with premature pubarche. A better delineation and description of HIDEA syndrome is required to determine whether hormonal dysfunction may be more significant than expected.

As another issue, in the case of ROHHAD, the risk of developing a neural crest tumor is well established and has been reported for 56% of ROHHAD patients (Bougnères et al., 2008; Harvengt et al., 2020). No data about tumor development in HIDEA patients has been published until now. Currently, this outcome is possibly lacking because some of the HIDEA patients are still young.

The first complete phenotypic description of patients with biallelic loss-of-function *P4HTM* gene variants was recently detailed in 2019 and extended in 2022 (Kaasinen et al., 2014; Rahikkala et al., 2019; Kraatari-Tiri et al., 2022). *P4HTM* was previously not integrated in the panels of the core genes list of intellectual disabilities. New versions of dedicated panels and updated algorithms for exome interpretations can currently highlight pathogenic variants in *P4HTM*. Therefore, it might be suggested to clinicians to ask for exome reinterpretations for patients with phenotypes similar to those with HIDEA or for patients with atypical ROHHAD presentation. Thanks to these technological improvements, more HIDEA patients will probably be diagnosed in the coming years, facilitating a better HIDEA phenotypic description.

In the same way, regarding the ROHHAD phenotypic description, a recent publication highlights the spectrum of ROHHAD patients without RO (Desse et al., 2022). An accurate phenotypic description of ROHHAD and ROHHAD-like patients is important to guide further research toward a better understanding of ROHHAD disease. The involvement of hypothalamic mechanisms seems to be the key point in this spectrum. In this respect, previous genetic and clinical comparisons have been described, for example, between ROHHAD and Prader–Willi syndrome (Barclay et al., 2018). It is currently unknown how to link Prader–Willi syndrome, central congenital hypoventilation syndrome (CCHS), or HIDEA to the spectrum of ROHHAD, but all these comparisons must be continuously studied in future clinical or molecular research studies.

At the molecular level, HIDEA is caused by biallelic pathogenic variants in the *P4HTM* gene. P4HTM is an endoplasmic reticulum transmembrane prolyl-4-hydroxylase (P4H) whose function is currently not exactly known (Rahikkala et al., 2019). P4Hs are enzymes that are not only involved in collagen synthesis but also in the regulation of the cellular response to hypoxia (Williams et al., 2007). A dysfunction in this last role seems to be a possible mechanism pathway interfering with mitochondrial function. Currently, intracellular mechanisms of the hypothalamic neurons are not completely understood, especially the possible role of the mitochondria in the regulation of metabolism and energy homeostasis (Jin and Diano, 2018). Apart from its role in the mitochondria, P4HTM has also been recently identified as a novel regulator of calcium signaling in astrocytes with potential disturbance to gliotransmission (Byts et al., 2021). Further knowledge on the role of P4HTM in central neurotransmission and hypothalamic neuronal connections is required to explore the possible mechanism of linking ROHHAD and HIDEA at the molecular level and investigate novel therapeutic research approaches in the future.

## Conclusion

Both ROHHAD and HIDEA are challenging diagnoses. In the case of clinical evidence of ROHHAD in a child with abnormal neurological development or eye abnormalities, the *P4HTM* gene should be systematically interrogated in addition to the *PHOX2B* analysis. In the future, a better delineation of the natural history of HIDEA is required to allow further comparisons between HIDEA and ROHHAD features, especially for dysautonomia and hypothalamic disturbances. The clinical similarities between the two syndromes should be better detailed to orient some molecular hypotheses. The investigation of P4HTM, an actor in neurotransmission, could bring new insight into the mechanism, possibly linking ROHHAD and HIDEA syndrome.

## Data Availability

The data sets for this article are not publicly available due to concerns regarding participant/patient anonymity. Requests to access the data sets should be directed to the corresponding author.

## Abbreviations

ROHHAD, rapid-onset obesity with hypothalamic dysfunction, hypoventilation, and autonomic dysregulation; ROHHAD (NET), rapid-onset obesity with hypothalamic dysfunction, hypoventilation, autonomic dysregulation, and neural crest tumor; HIDEA, hypotonia, hypoventilation, impaired intellectual development, dysautonomia, epilepsy, and eye abnormalities.
